# Geolocation data of irrigation network in water user association's operation area under community-based and provider-based network governance

**DOI:** 10.1016/j.dib.2020.106168

**Published:** 2020-08-12

**Authors:** Ahmad Fatikhul Khasan, Mohammad Rondhi, Yasuhiro Mori, Takumi Kondo

**Affiliations:** a Department of Agribusiness, University of Jember, Jember 68121, Indonesia; b Doctoral Program of Agricultural Science, University of Jember, Jember 68121, Indonesia; c Department of Sustainable Agriculture, Rakuno Gakuen University, Ebetsu, Hokkaido 069-8501, Japan; d Research Faculty of Agriculture, Hokkaido University, Sapporo 060-8589, Japan

**Keywords:** WUAs governance, Provider-based network, Community-based network, Geolocation data, Indonesian irrigation

## Abstract

Massive irrigation development has influenced the way farmers govern water user associations (WUAs) in Klambu Wilalung Irrigation District in Central Java, Indonesia. Recently, farmers adopt community-based (*swakelola*) and provider-based (*lelang*) network governance to manage the WUAs. The physical conditions of the irrigation network have become the primary driver of WUA's governance selection. This article provides geolocation data of irrigation network in the operation area of WUAs under community-based and provider-based network governance. The irrigation network consisted of two components, irrigation canal, and structure. Irrigation canal divided into the primary and secondary canal. The data cover GPS Tracks coordinates for 75 secondary canals and seven primary canals. Meanwhile, irrigation structures were grouped into main and support structures and consists of 12 types of structure. The data covers the GPS waypoint coordinates for 194 irrigation structures. The data also provide basic information of 34 WUAs applying community-based (11 WUAs) and provider-based (23 WUAs) network governance. WUAs basic information obtained from a survey conducted in 2016 and covers information on the type of governance, number of board, number of member farmers, size of operation area, and administrative location of each WUAs. Finally, the data is useful in planning research aimed to explain the role of irrigation infrastructure in shaping WUAs governance. Also, the information is crucial to planning a field survey in the area of the Klambu Wilalung Irrigation District in Central Java.

**Specifications Table**

**Table:** 

Subject	Agricultural science
Specific subject area	Irrigation, water user associations governance
Type of data	Table Graph ESRI shapefiles
How data were acquired	GPS coordinates acquired from irrigation officials of the Klambu Wilalung Irrigation District. Then, we used the coordinate data to create the shapefile (.shp) of the irrigation network. QGIS and Google Earth software was used to develop the shapefiles.
Data format	Raw Analyzed
Parameters for data collection	We select all irrigation networks (irrigation canals and structures) located in the operation area of WUAs, adopting community-based and provider-based network governance in the Klambu Wilalung Irrigation District in Central Java, Indonesia.
Description of data collection	The data collection consists of three stages. 1 We enumerate all WUAs in Klambu Wilalung. In this stage, we collect basic information on administrative location, the type of governance, number of the board members, WUAs operation area, and number of member farmers. 2 We collect GPS coordinates of the entire irrigation network from irrigation officials. Then we plotted the coordinates using QGIS and locate each irrigation facility (canals and structure) to its corresponding WUA operation area. 3 Then, we create the shapefiles (.shp) for each irrigation facility in each WUAs operation area.
Data source location	Institution: Klambu Wilalung Irrigation District City/Town/Region: Grobogan and Kudus Regency (*Kabupaten)*, Province of Central Java Country: Indonesia Latitude and longitude (and GPS coordinates, if possible) for collected samples/data: The GPS coordinates of each WUAs are provided in the dataset with this data article.
Data accessibility	The data is provided with the article.
Related research article	M. Rondhi, A. F. Khasan, Y. Mori, T. Kondo, Absence of legislation and the quest for an effective mode of governance in agricultural water management: An insight from an irrigation district in central java, Indonesia *, Irrig. Drain. (2020) 1–12. https://doi.org/10.1002/ird.2450 [1]

Value of the Data

• The geolocation data of the irrigation network in the WUA operation area facilitate accurate identification of the location of irrigation infrastructure for each WUA. It simplifies the process of tracking and marking irrigation construction that is central in the study of WUA under community-based (*swakelola*) and provider-based (*lelang*) governance in the Klambu Wilalung Irrigation District, Central Java, Indonesia.
• Researchers working on common-pool resource theory, especially on network governance, who are looking for a new case study might use this data as a guide for their new survey. Irrigation practitioners in Indonesia working on strengthening WUA's organizational capacity might use this data to locate an exemplary WUA model for WUA in other regions or provinces.
• The spatial data of the Klambu Wilalung irrigation network is useful to analyze the motive of WUA's governance selection. This data is crucial in answering the hypothesis of whether physical conditions of irrigation networks affect WUA's governance selection.
• Historically, WUA's governance changed as political and social changes occurred. This spatial data is based on the situation in 2015, representing the period from 1990 to 2015. This data is useful to build a historical construct of WUA's governance in future researches.
• The data serves as a reference to identify the interrelationship between the presence or absence of irrigation infrastructure and the selection of WUA governance.
• The shapefiles can be used to create the irrigation map on each WUA's operation area.

## 1 Data Description

There are three types of data in this data article, (1) the dataset, (2) the map of WUA's operation area under provider-based and community-based network governance, and (3) the shapefiles of irrigation network in each village.

(1) *The dataset*

The dataset is in Microsoft excel format. It consists of two worksheets, (1) the WUA and (2) geolocation_data worksheet. The first worksheet provides information on the basic characteristics of WUA and contains twelve variables. The second worksheet provides geolocation data of the irrigation network in each village. Table 1 presents the descriptions of variables in WUA and geolocation_data worksheets.

**Table 1:** Descriptions of variables in the WUA worksheet

No	Name	Description	Code	Measure	Unit	Source
1	Identity	Unique ID to each WUA in the Klambu Wilalung Irrigation District	*ID*			Enumeration
2	Official name	The official name of WUA as registered in the irrigation offices of Kudus and Grobogan Regency	*name*			Enumeration
3	Province^a^	The province (Provinsi) in which WUA situated.	*Prov*			Enumeration
4	Regency	The regency/city (*Kabupaten/Kota*) in which WUA situated.	*Reg*			Enumeration
5	District	The district (*kecamatan*) in which WUA situated.	*Dis*			Enumeration
6	Village	The village (*desa*) in which WUA is situated.	*village*			Enumeration
7	Hamlet^b^	The hamlet (RW) in which WUA situated	*hamlet*			Enumeration
8	Neighborhood^b^	The neighborhood (RT) in which WUA situated	*neighborhood*			Enumeration
9	Governance	The type of WUA governance (1=provider-based network, 2=community-based network)	*Gov*	Nominal		Enumeration
10	Area	WUA's operation area based		Scale	ha	Enumeration
11	Board Member	The number of WUA's board member including the chairman	*board*	Scale	person	Enumeration
12	Member farmer	The number of farmers belongs to the WUA	*member*	Scale	person	Enumeration

Note:

a The province is the first-level administrative region in Indonesia, followed by regency (Kabupaten/Kota), district (kecamatan), village (desa/kelurahan). Province, district, and village headed by official winning the largest popular vote. While kecamatan, although larger than village, led by official selected by Reagent (Bupati) and under district command.

b Hamlet and neighborhood are the administrative areas under village and village command.

The geolocation_data worksheet contains the coordinates of each irrigation components in the Klambu Wilalung Irrigation District. There are two irrigation network components, irrigation structure, and canal. Table 2 provides variable descriptions in the geolocation_data worksheet.

(1) *The map of irrigation networks*

**Table 2:** Descriptions of variables in the geolocation_data worksheet

No	Name	Description	Code	Measure	Unit	Source
1	Identity	Unique ID to each irrigation network component. The ID is the official nomenclature from Klambu Wilalung Irrigation District.	*ID*			Official data
2	Name	The name of each irrigation network component	*name*			Official data
3	Type^a^	The type of component in the irrigation network (1=structure, 2=canal)	*type*	Nominal		Official data
4	Status	The status of irrigation network components. For structures (1=main structures, 2=supporting structures). For canals (1=primary canal, 2=secondary canal).	*stat*	Nominal		Official data
5	X Coordinate^b^	The Easting coordinate in UTM Projection System	*X*			Official data
6	Y Coordinate^b^	The Northing coordinate in UTM Projection System	*Y*			Official data
7	WUA	The WUA for which each irrigation network components situated				Enumeration
8	Village	The village for which each irrigation network components situated				Enumeration

Note:

a The coordinates povided only for irrigation structure

b The geodetic datum for UTM coordinates is WGS 84/UTM Zone 49 S

**Fig. 1:**
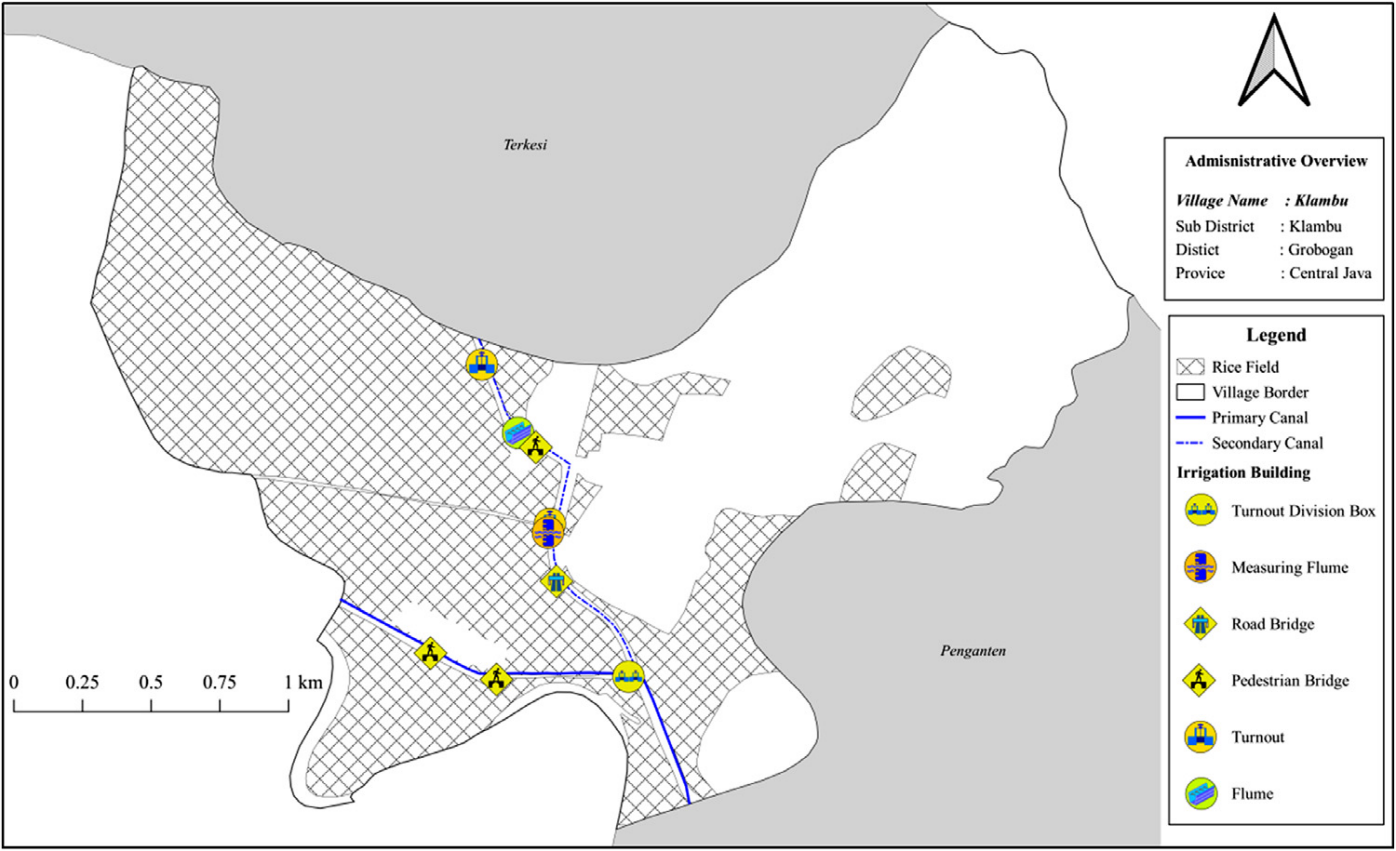
The irrigation network in Klambu village

**Fig. 2:**
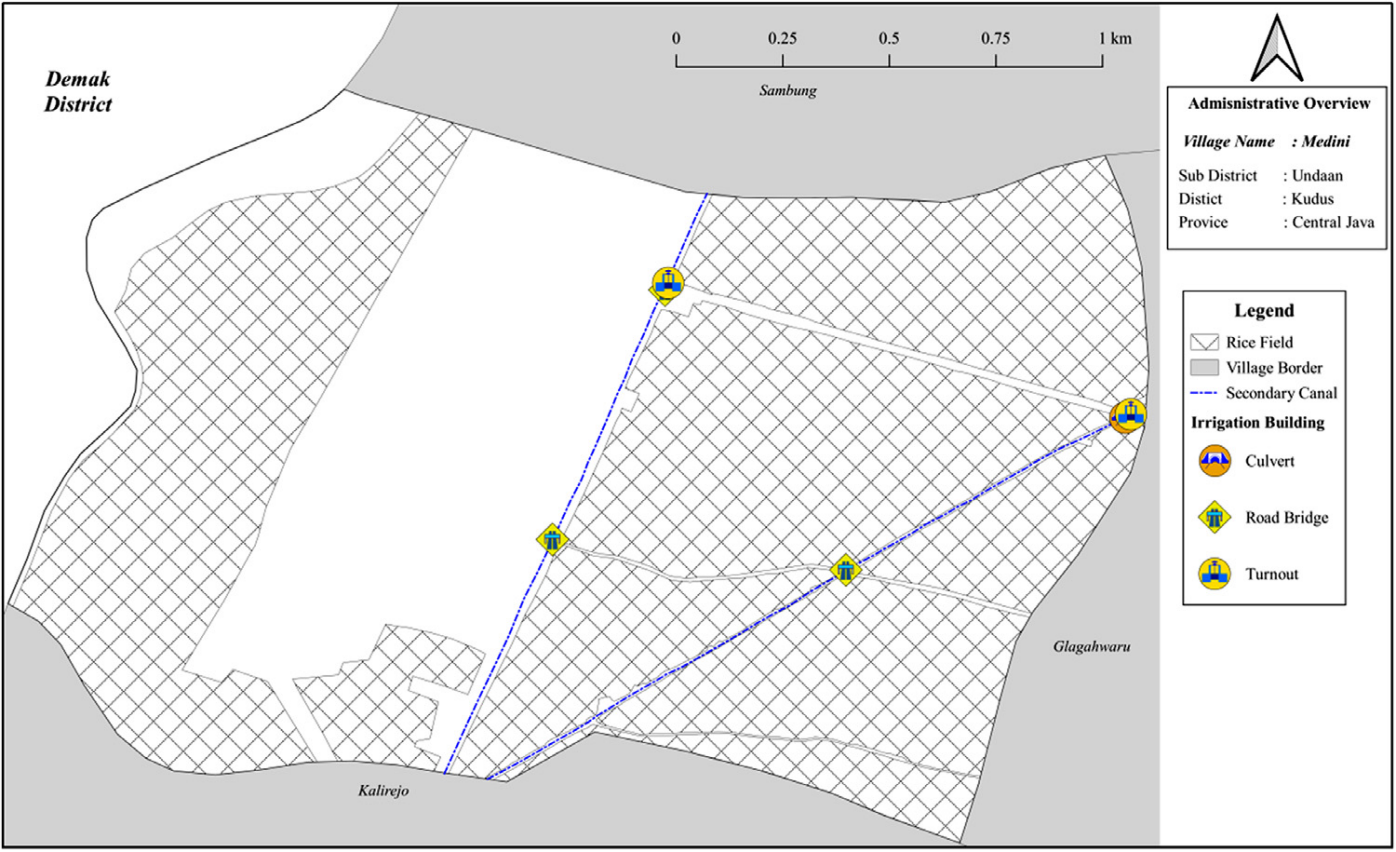
The irrigation network in Medini village

The following data is the map for the village in which the WUA adopted provider-based and community-based governance. The figures provided in this article are examples from two villages, Klambu (Fig. 1) and Medini (Fig. 2) villages. Klambu has one WUA (*Mbangun Tani*) and adopted a *community-based network* as its mode of governance. On the other hand, Medini has three WUAs (*Kayu Urip, Kandang Rejo, Pingkuk Mulyo*), which managed under *provider-based network* governance. The complete list of WUA is provided in the dataset. The irrigation map similar to Fig. 1 and Fig. 2 are uploaded with this article.

(1) *The shapefiles of irrigation networks*

The shapefiles from which irrigation map was created are also uploaded with this article. The shapefiles have been grouped by village. A catalouge containing detailed information for each shapefiles is provided in Microsoft Excel format (Shapefiles catalouge.xlsx). The catalouge consists of two worksheet, the *line_polygon* and *point* worksheets. The *line_polygon* worksheet contains information on the shapefiles of village border, Irrigation canals, and irrigated farmland. Table 3 provides the descriptions of the shapefiles.

**Table 3:** Descriptions of the shapefiles in the *line_polygon* worksheet

No	Name	Description	Code	Geometry point
1	Primary canal	Irrigation canal connected directly to a water source	*Primcanal_name*	line
2	Secondary canal	Irrigation canal below the primary canal	*Seccanal_name*	line
3	Irrigated Farmland	The area of irrigated farmland in each village	*farmland_village*	polygon
4	Village Border	The administrative border of each village	*villborder_village*	polygon

The *point* worksheet contains information on the shapefiles of irrigation structures. The irrigation structure in each village are grouped into one shapefile. Table 4 describes each structure in the shapefiles.

**Table 4:** Descriptions of the second worksheet in shapefiles catalouge

No.	Name	Indonesian Name	Description
1	Division box	Bagi	Irrigation structure used to divide water between two or more canals
2	Division-turnout box	Bagi-sadap	Irrigation structure used to divide the water and divert water to subsequent canals
3	Turnout	Sadap	Irrigation structure used to divert water to the smaller canal
4	Culvert	Gorong-gorong	Irrigation structure used to carry water across the road and located underground
5	Cross-Culvert	Gorong-gornong Silang	Similar to general culvert, the only different is in placement arrangement.
6	Pedestrian Bridge	Jembatan Orang	Bridge over irrigation canal (only for pedestrian)
7	Road Bridge	Jembatan Desa	Bridge over irrigation canal (for vehicles)
8	Flume	Talang	Irrigation structure used to carry water across gullies or ravines
9	Measuring Flume	Bangunan Ukur	Irrigation structure used to measure water flow
10	Siphon	Siphon	Irrigation structure used to transfer water over a barrier
11	Oncoran	Oncoran	Irrigation structure used to transfer water from sewer to farmland
12	Side Weir	Pelimpah Samping	Irrigation structure used to drain water from the central canal when the water surface exceeds the maximum level

Note: The geometry point of all irrigation structure is point. All irrigation structure in each village are grouped into one shapefile. The shapefile name format is *plot_village name.*

## 2 Experimental Design, Materials and Methods

### 2.1 Area of data collection

**Fig. 3:**
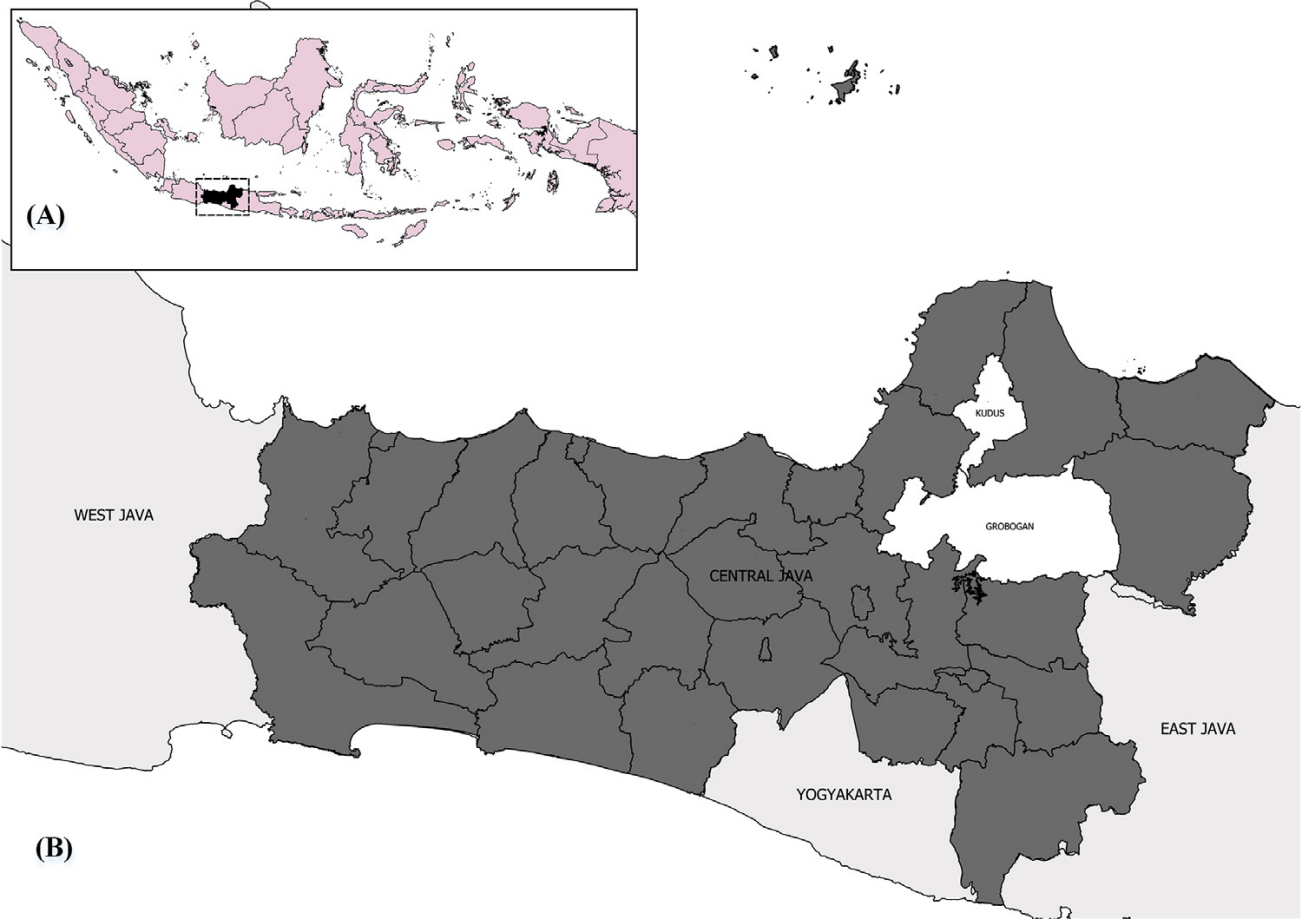
The location of data collection (A) Province of Central Java relative to Indonesia, (B) Grobogan and Kudus Regency relative to Province of Central Java (Area highlighted in white).

The data presented here represent Klambu Wilalung Irrigation District (KWID). KWID located in Grobogan and Kudus Regency, Province of East Java, Indonesia. The data cover the area of 20 villages and 34 WUAs. Fig. 3 shows the location of KWID.

The data covers twenty villages and 34 WUAs. Four villages located in Grobogan and have four WUAs. The remaining sixteen villages are situated in Kudus and have 30 WUAs. There are 24 WUAs adopted provider-based network governance, and 11 WUAs took community-based network governance.

### 2.2 Data collection

There are two types of data collected, (1) the WUAs characteristics and (2) the coordinates of irrigation network in KWID. We gathered the first data through enumeration to all WUAs chairman in KWID. This process is similar to the enumeration process in our previous data article [2]. The purpose of this enumeration is to define the location of each WUA and its corresponding operation area. We also collected information on the essential characteristics of WUA, such as presented in Table 1. The result of this enumeration was utilized as a reference in conducting the comprehensive survey presented in our paper [1].

**Fig. 4:**
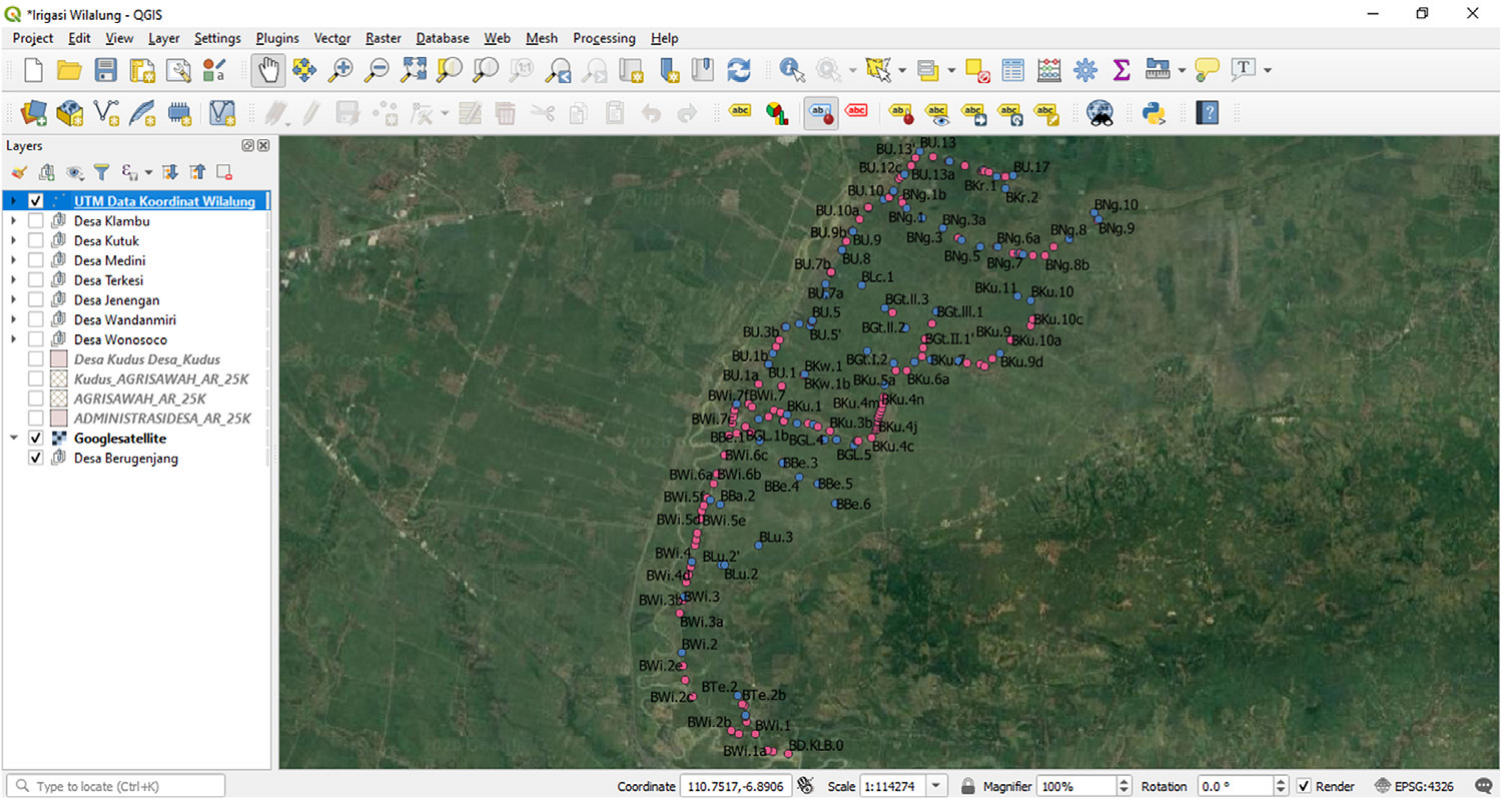
The plotting of original coordinates data in QGIS

We obtained the coordinates of irrigation networks from irrigation officials in KWID. We then used the coordinates to locate the irrigation networks—all the spatial analysis performed using QGIS software (version 3.12.3). The coordinates data are in UTM format using WGS 84/UTM Zone 49 S. Fig. 4 shows the plotting of the original coordinates data. We then used the original coordinates data to identify irrigation structures and canals. We use Google Satellite images to verify the coordinate points of KWID. We imported the Google Map layer to the QGIS using XYZ tiles.^1^ The official village administrative border and farmland layer obtained from the Indonesian Geospatial Information Agency.^2^

**Fig. 5:**
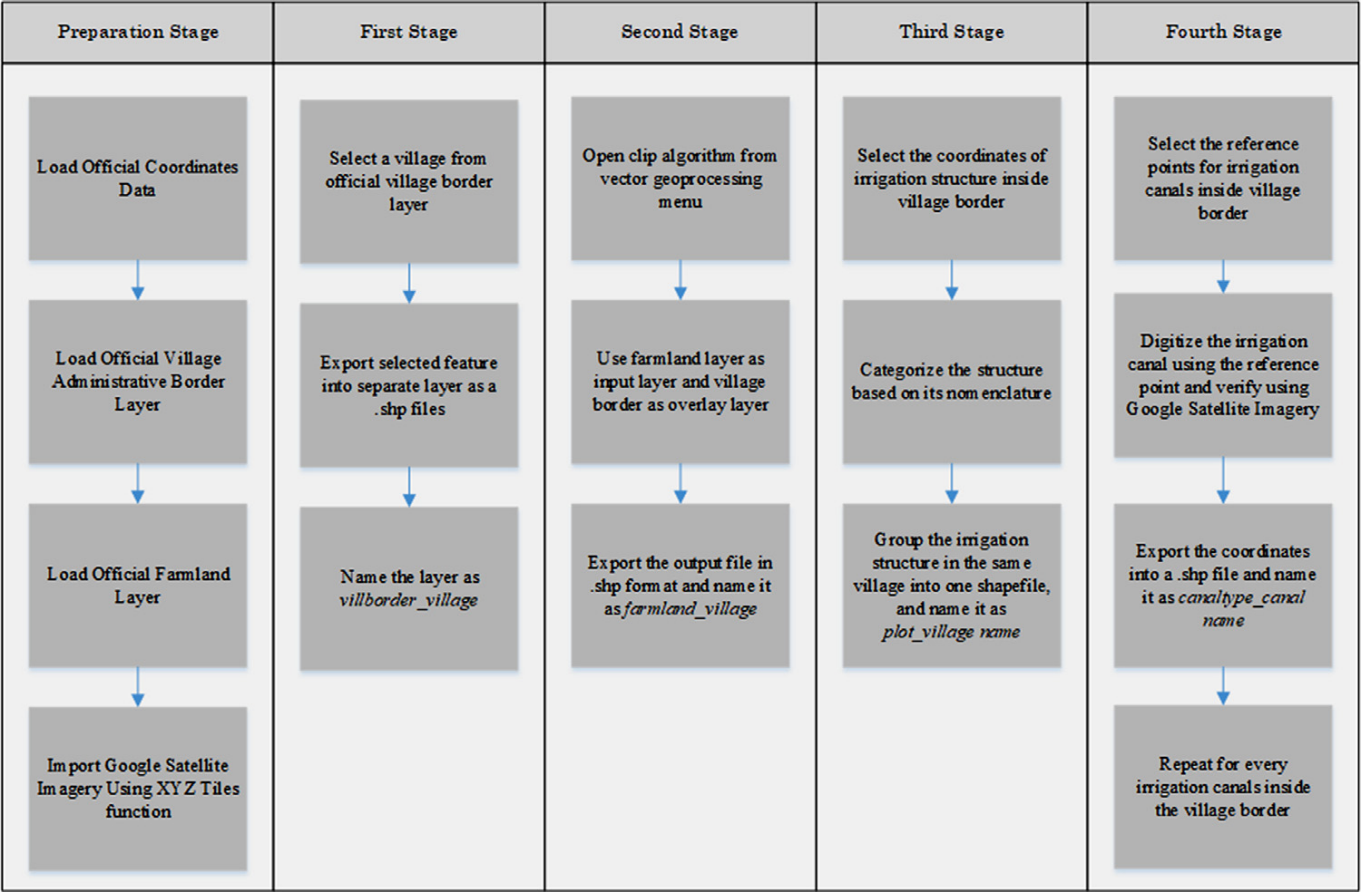
The spatial analysis workflows

The spatial analysis to create shapefiles data consists of four stages. In the first stage, we define the village boundary to locate the operation area of each WUA. The official administrative village border was used in this stage. In the second stage, we select the irrigated farmland in a particular village. We use the *clip* algorithm of QGIS to create a new layer of farmland specific to a specific village. The official farmland area in Kudus and Grobogan was used as the input files. And the village border was used as the overlay layer. In the third stage, we digitized the irrigation structures located in the village. We grouped each structure type into one shapefiles. Finally, in the fourth stage, we digitized the irrigation canals. The official coordinate data only provide the reference point (the starting and ending point) for each irrigation canal. We then created a new line connecting these reference points. Google Satellite Imagery was used to verify the line remotely. Fig. 5 shows the workflow of spatial analysis.

## Ethics Statement

Implied informed consent was obtained from all participant in this study.

## Credit Author Statement

Ahmad Fatikhul Khasan: Conceptualization, Methodology, Software, Writing original draft. Mohammad Rondhi: Conceptualization, Investigation, Review, Supervision, Writing original draft. Yasuhiro Mori: Investigation, Visualization. Takumi Kondo: Supervision, Investigation, Funding.

## Funding

This work was supported by JSPS KAKENHI, Grant Number 18K05839.

## Declaration of Competing Interest

The authors declare no competing interest.

## Appendix Supplementary materials

Image, application 1

Image, application 2

Image, application 3

## Footnotes

1 The URL for XYZ tiles function is provided as Supplementary Material with this data article.

2 URL: https://tanahair.indonesia.go.id/portal-web
