# Supplementary figures and images for: Geolocation data of irrigation network in water user association's operation area under community-based and provider-based network governance
Source: Data Brief. 2020 Aug 12;32:106168. doi: 10.1016/j.dib.2020.106168 (PMC7452706; doi:10.1016/j.dib.2020.106168)

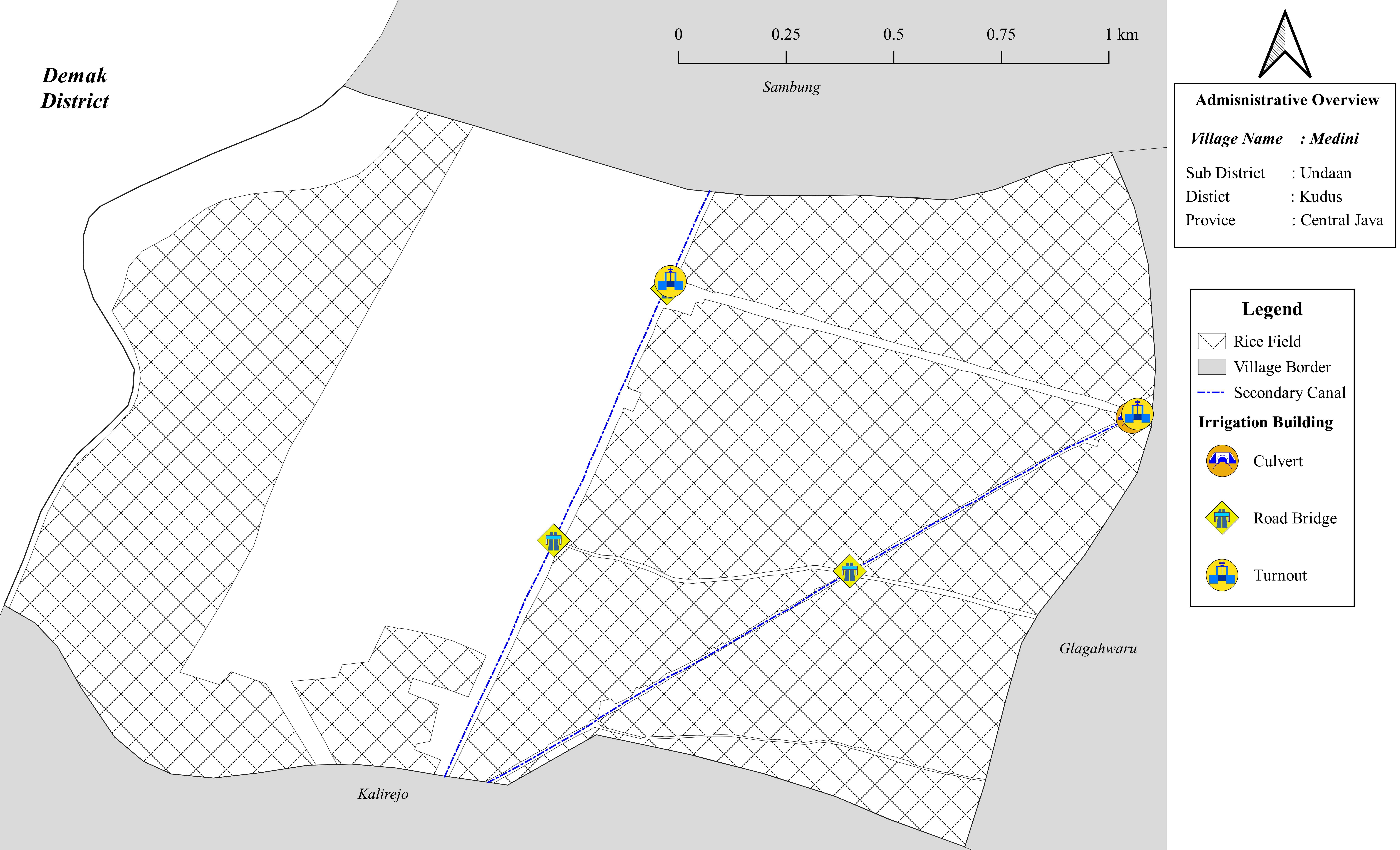

Supplement: Supplementary file 2 [file mmc2.zip › Supplementary data files/2. Irrigation Map/1. Kudus/1. Medini.png]

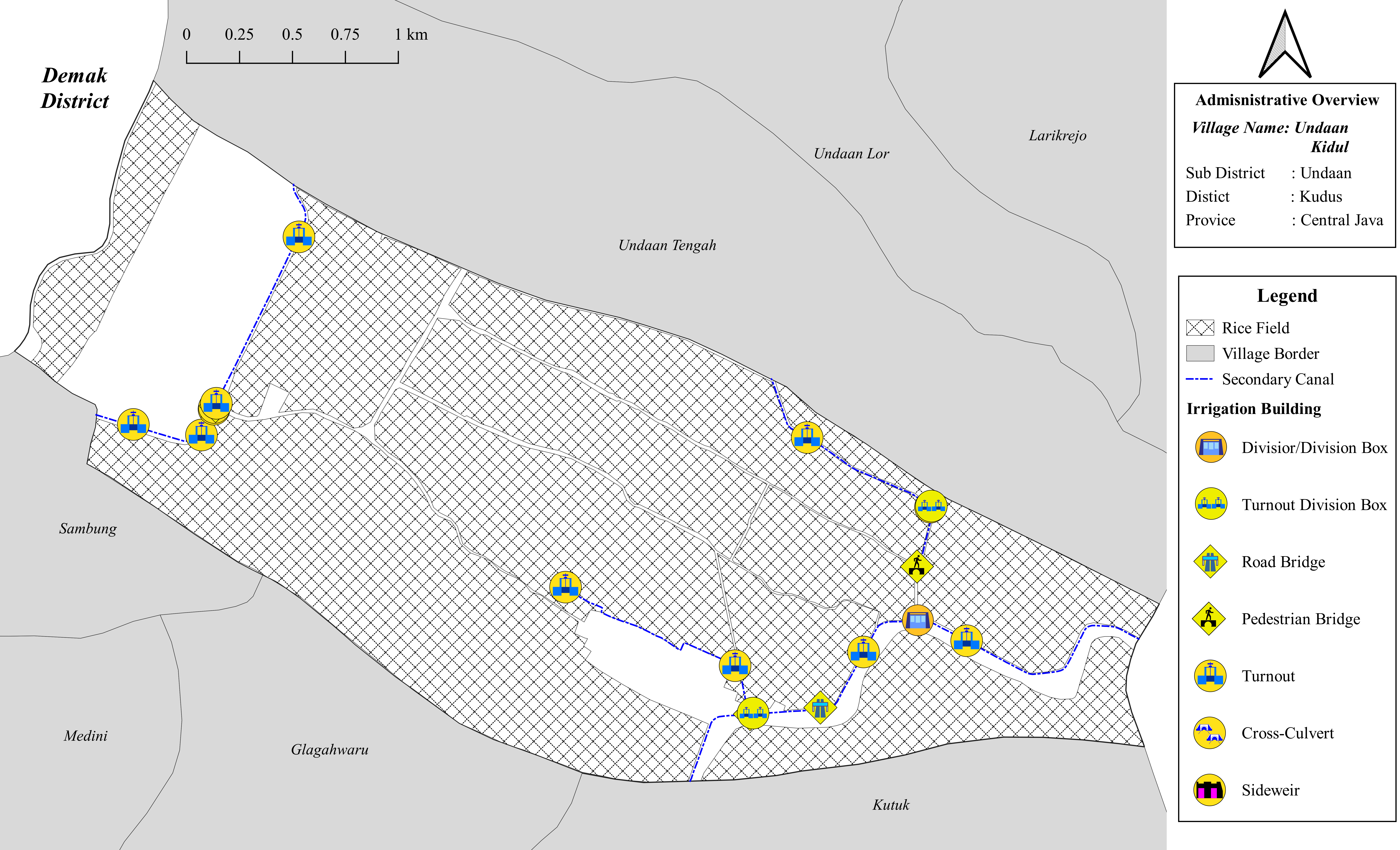

Supplement: Supplementary file 2 [file mmc2.zip › Supplementary data files/2. Irrigation Map/1. Kudus/10. Undaan Kidul.png]

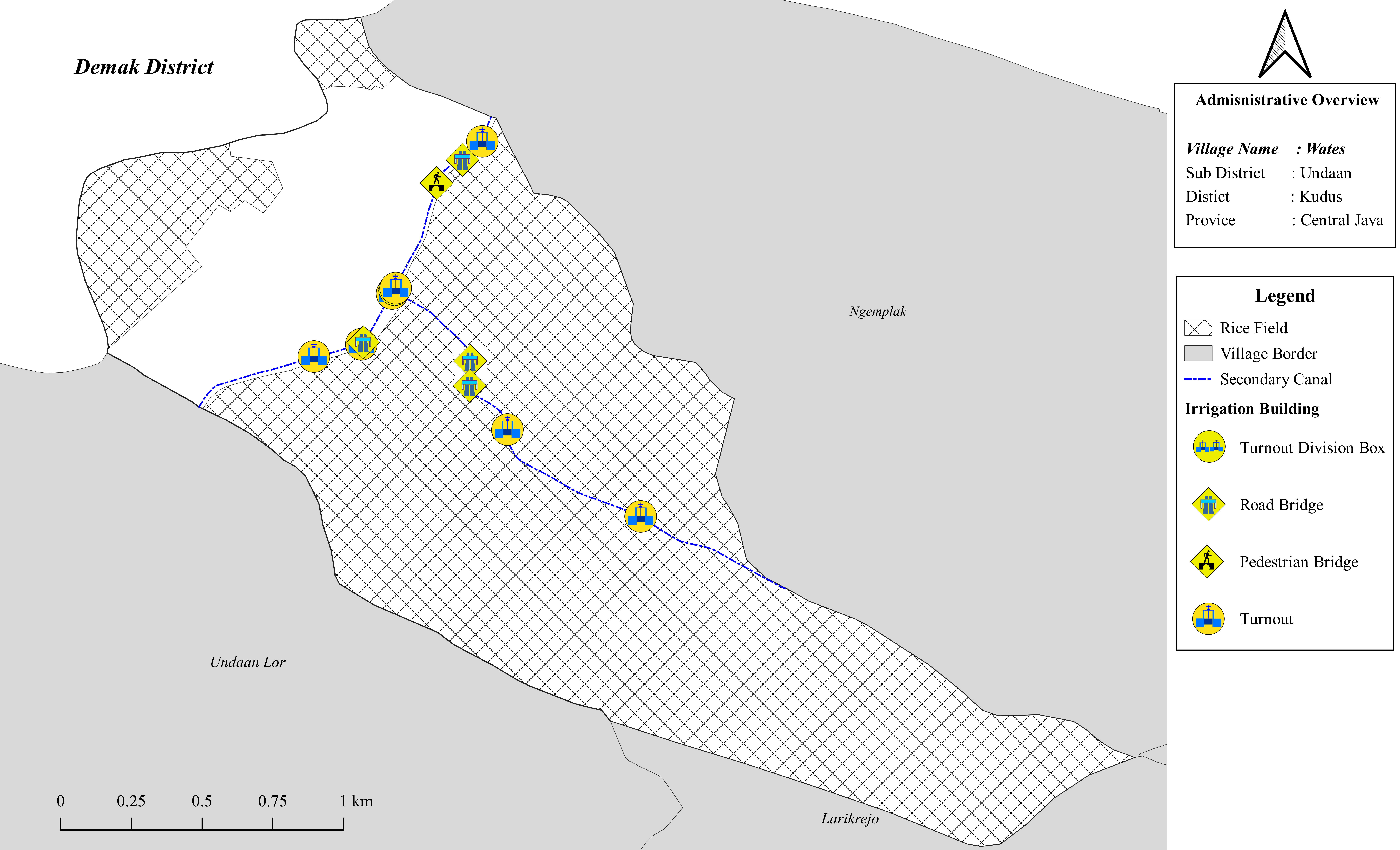

Supplement: Supplementary file 2 [file mmc2.zip › Supplementary data files/2. Irrigation Map/1. Kudus/11. Wates.png]

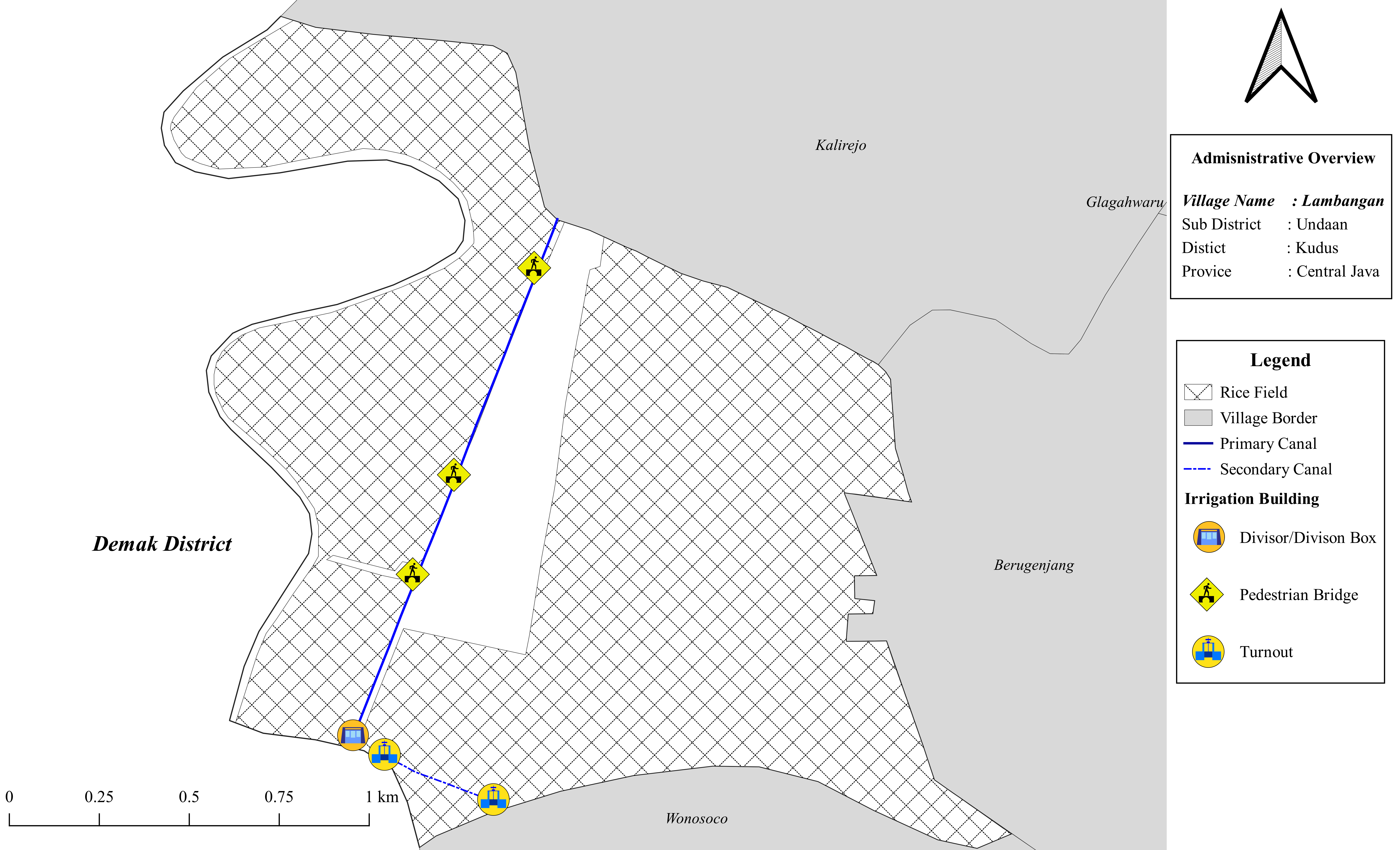

Supplement: Supplementary file 2 [file mmc2.zip › Supplementary data files/2. Irrigation Map/1. Kudus/12. Lambangan.png]

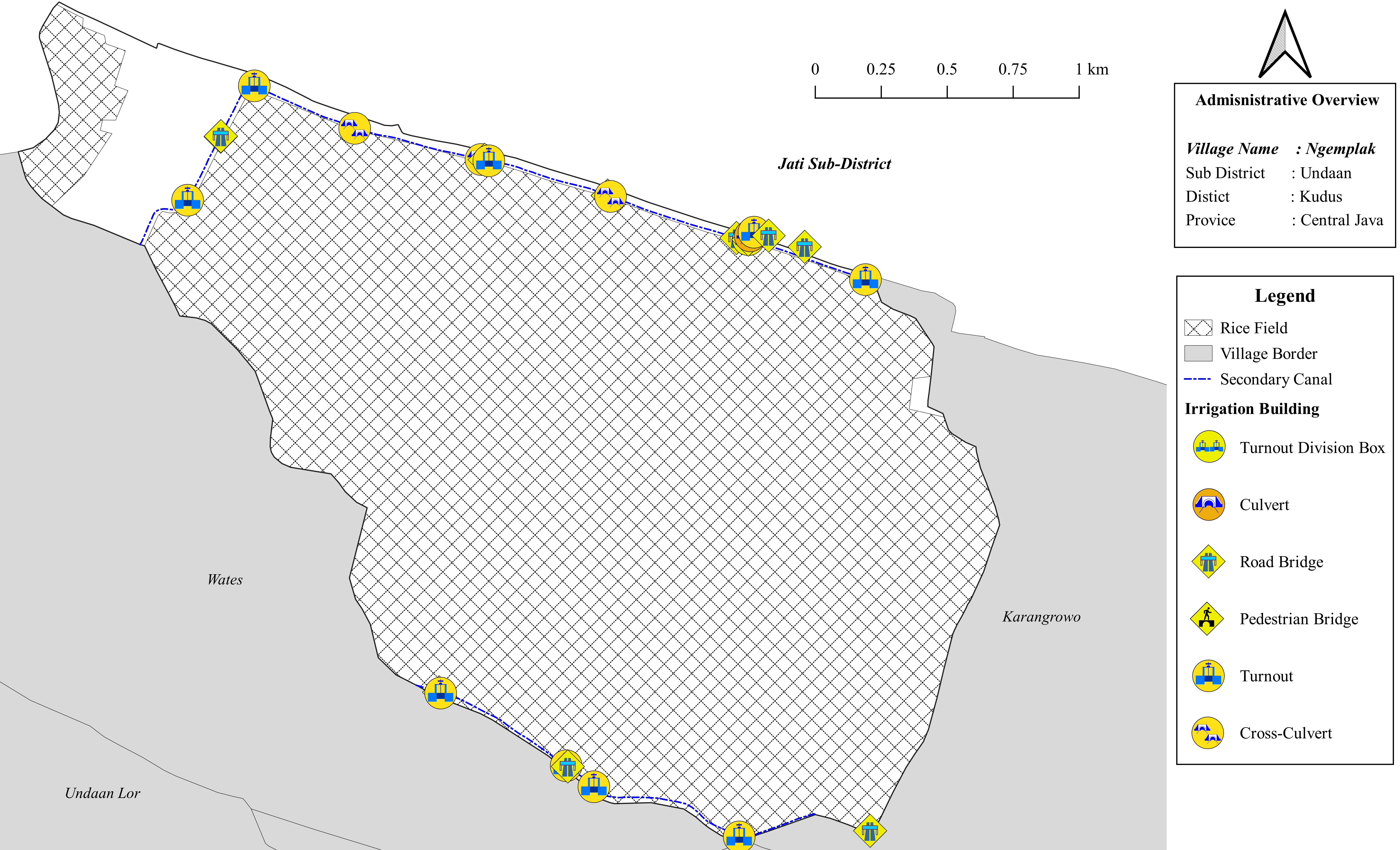

Supplement: Supplementary file 2 [file mmc2.zip › Supplementary data files/2. Irrigation Map/1. Kudus/13. Ngemplak.png]

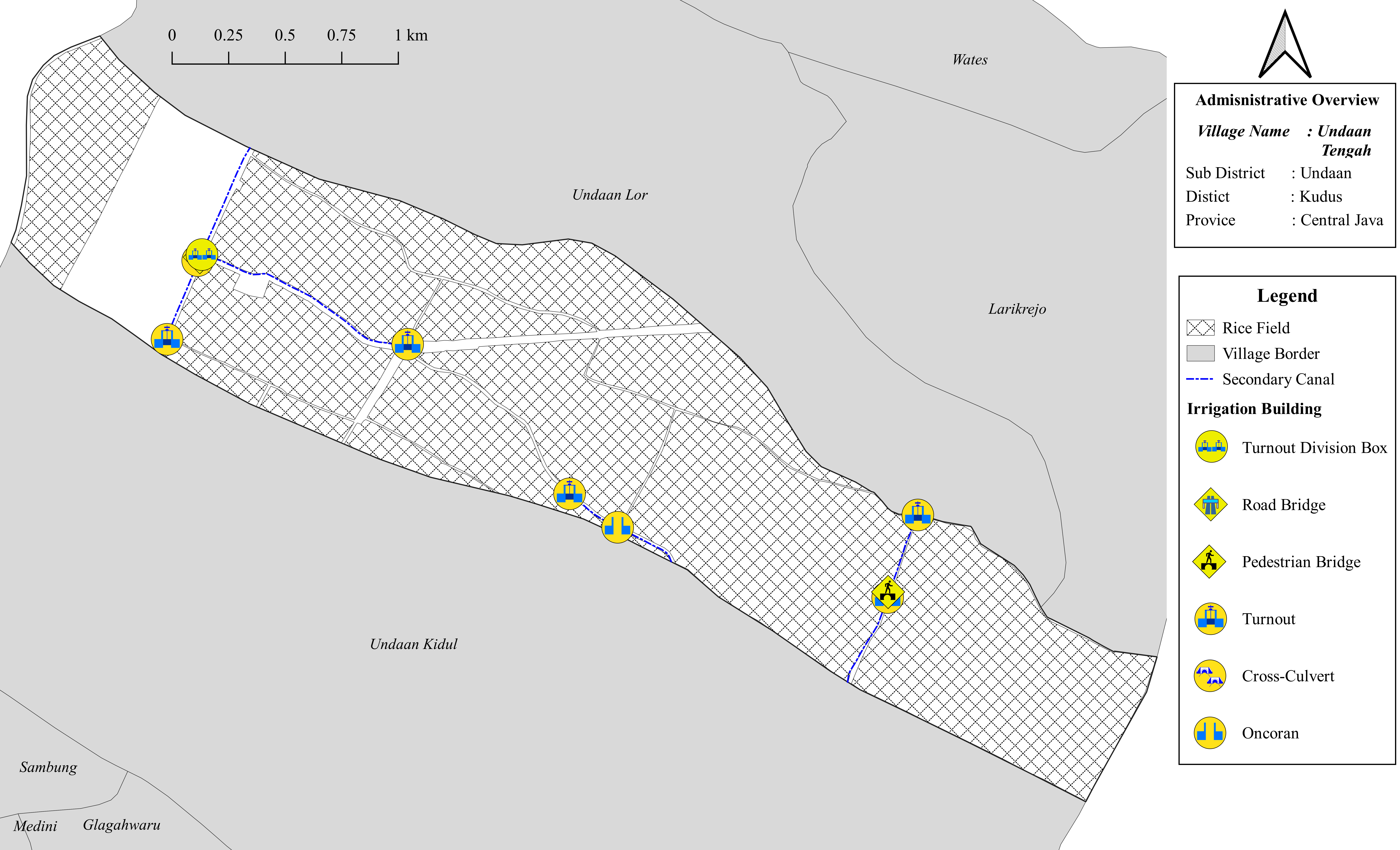

Supplement: Supplementary file 2 [file mmc2.zip › Supplementary data files/2. Irrigation Map/1. Kudus/14. Undaan Tengah.png]

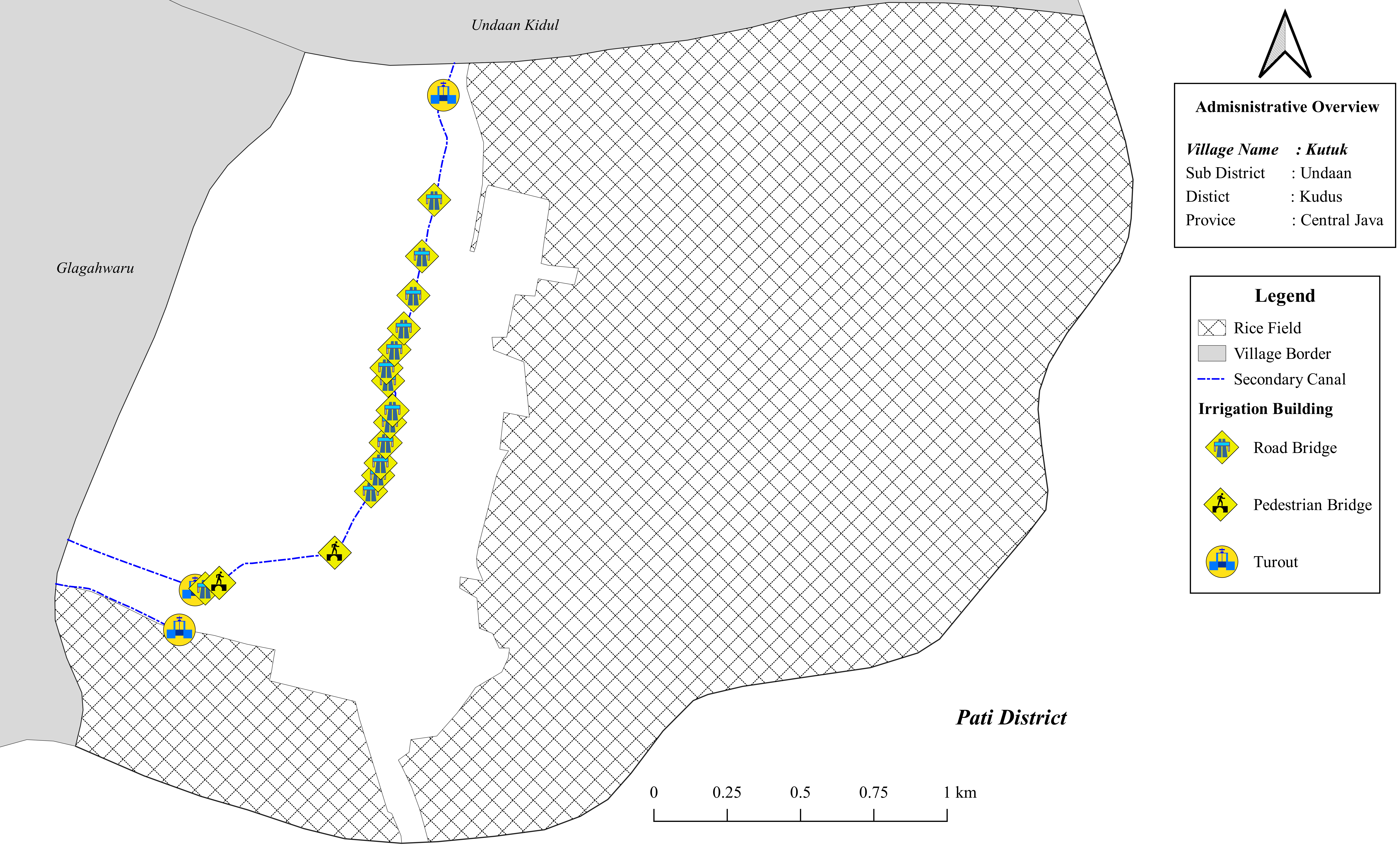

Supplement: Supplementary file 2 [file mmc2.zip › Supplementary data files/2. Irrigation Map/1. Kudus/2. Kutuk.png]

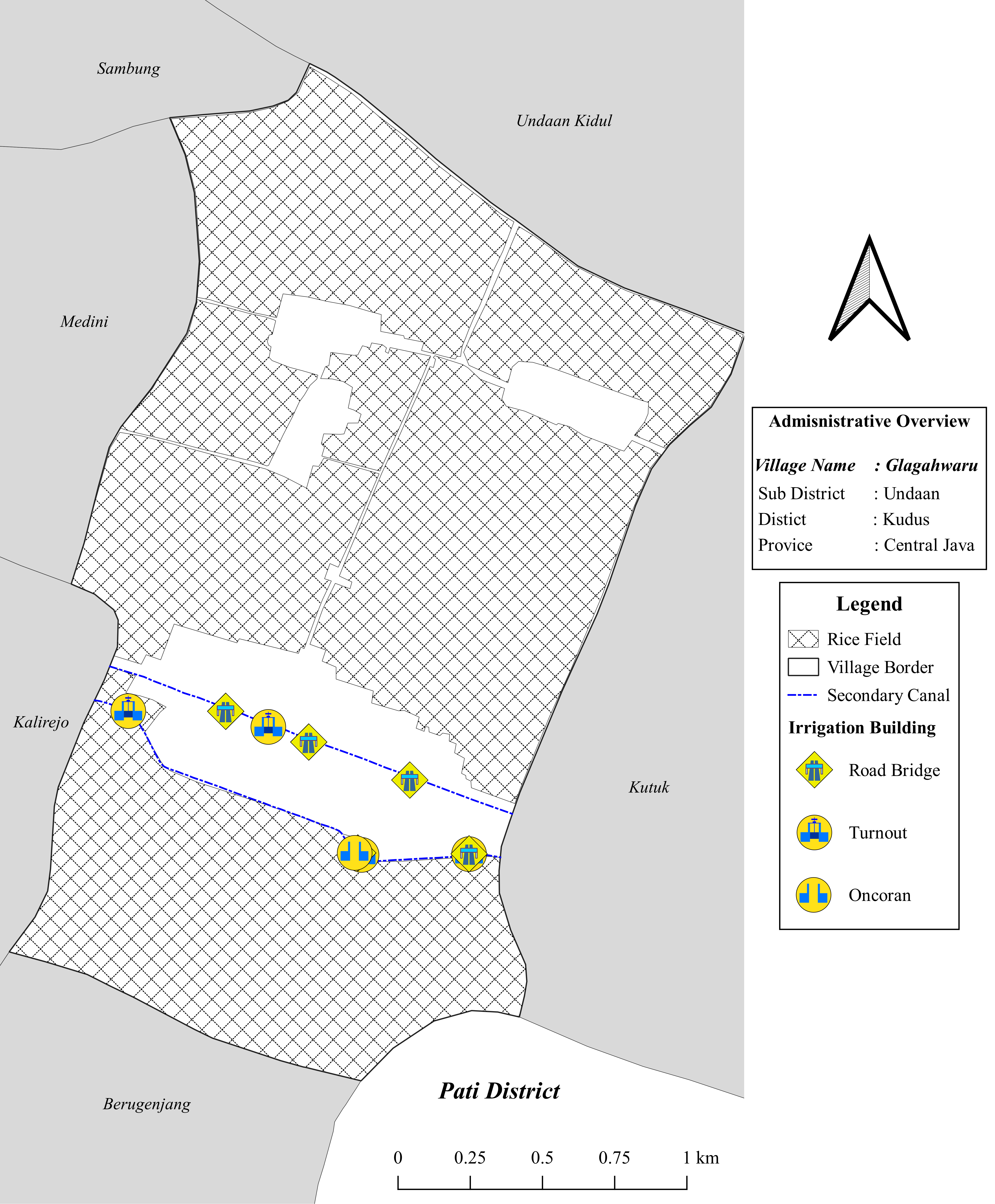

Supplement: Supplementary file 2 [file mmc2.zip › Supplementary data files/2. Irrigation Map/1. Kudus/3. Glagahwaru.png]

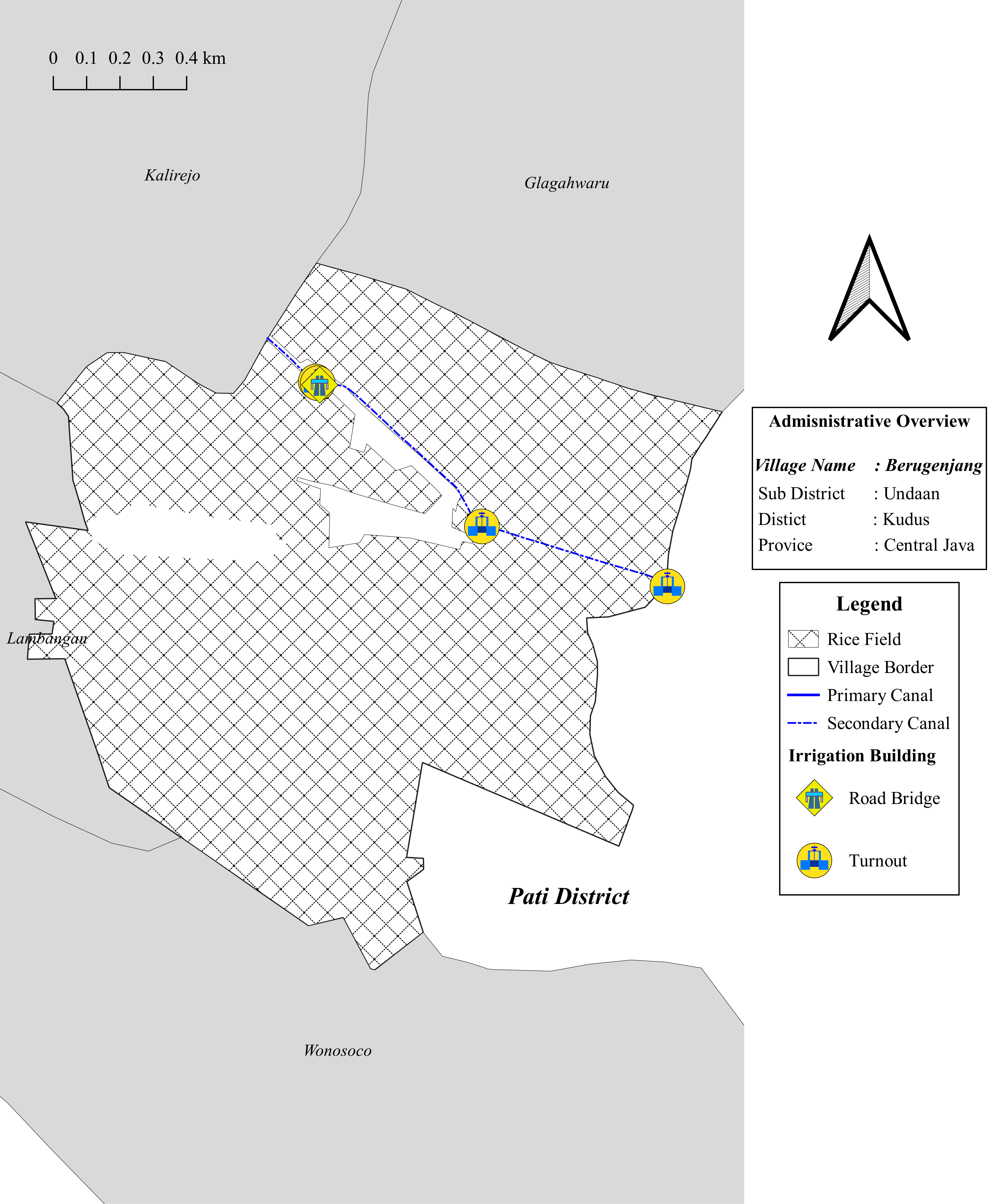

Supplement: Supplementary file 2 [file mmc2.zip › Supplementary data files/2. Irrigation Map/1. Kudus/4. Berugenjang.png]

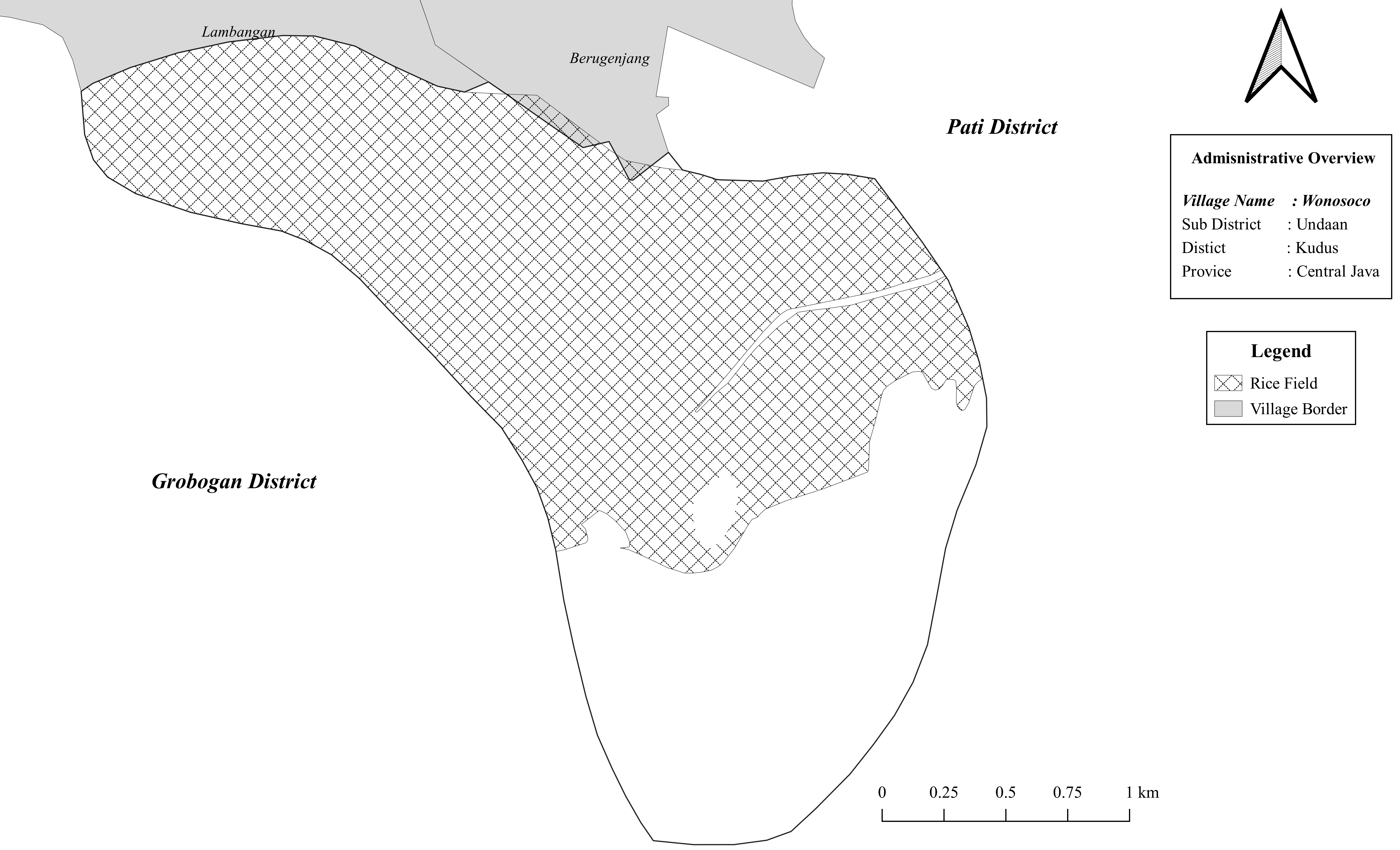

Supplement: Supplementary file 2 [file mmc2.zip › Supplementary data files/2. Irrigation Map/1. Kudus/5. Wonosoco.png]

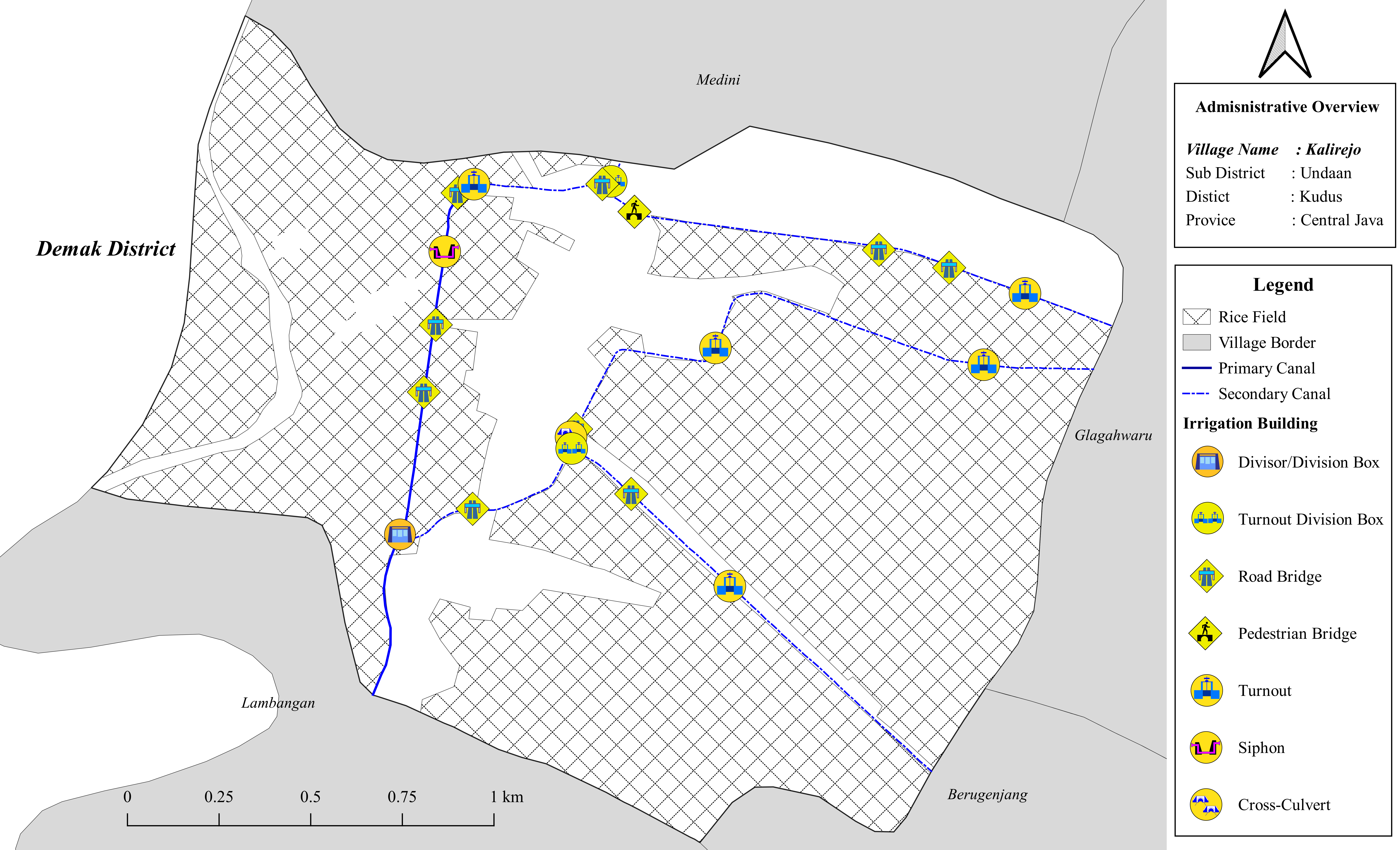

Supplement: Supplementary file 2 [file mmc2.zip › Supplementary data files/2. Irrigation Map/1. Kudus/6. Kalirejo.png]

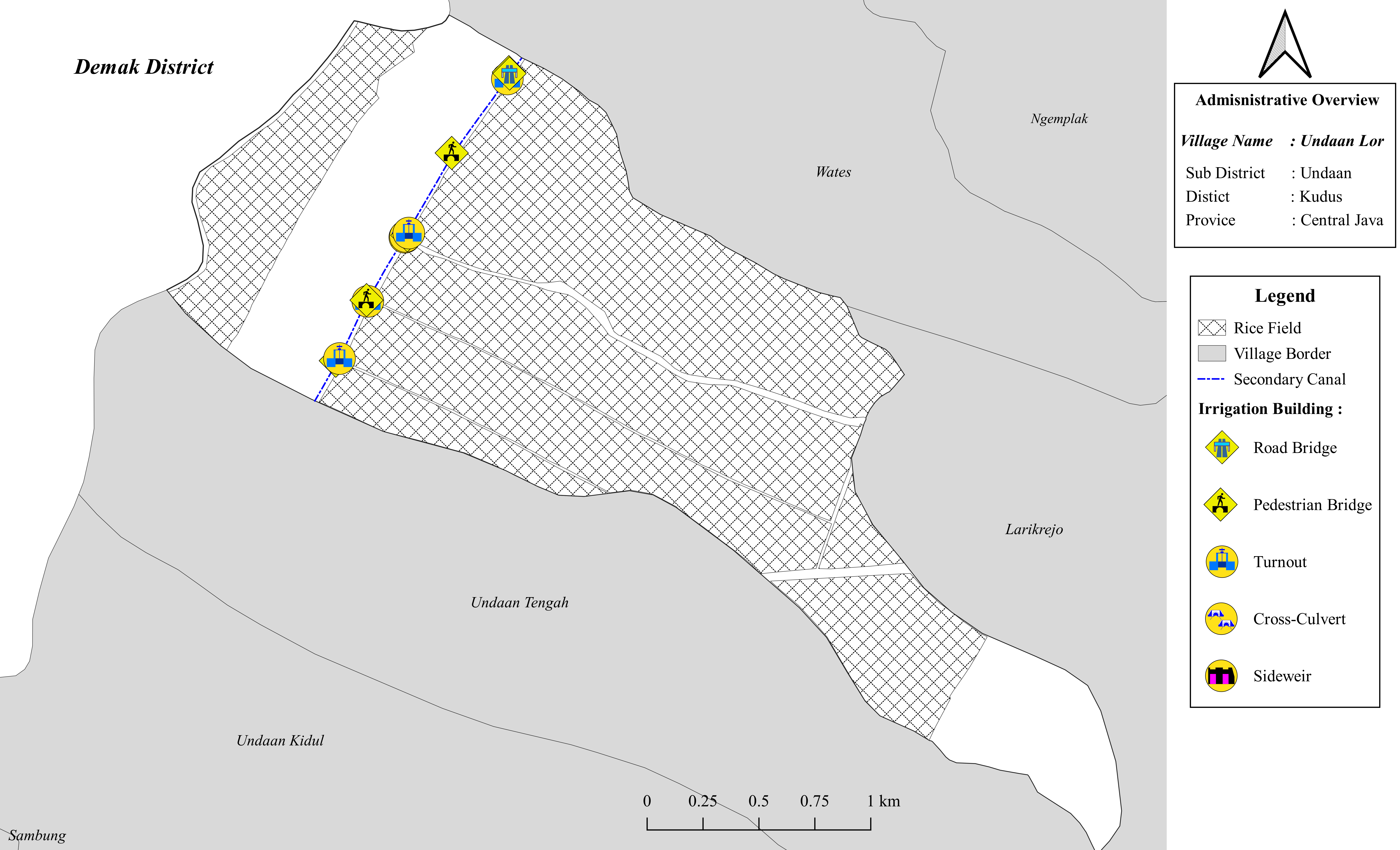

Supplement: Supplementary file 2 [file mmc2.zip › Supplementary data files/2. Irrigation Map/1. Kudus/7. Undaan Lor.png]

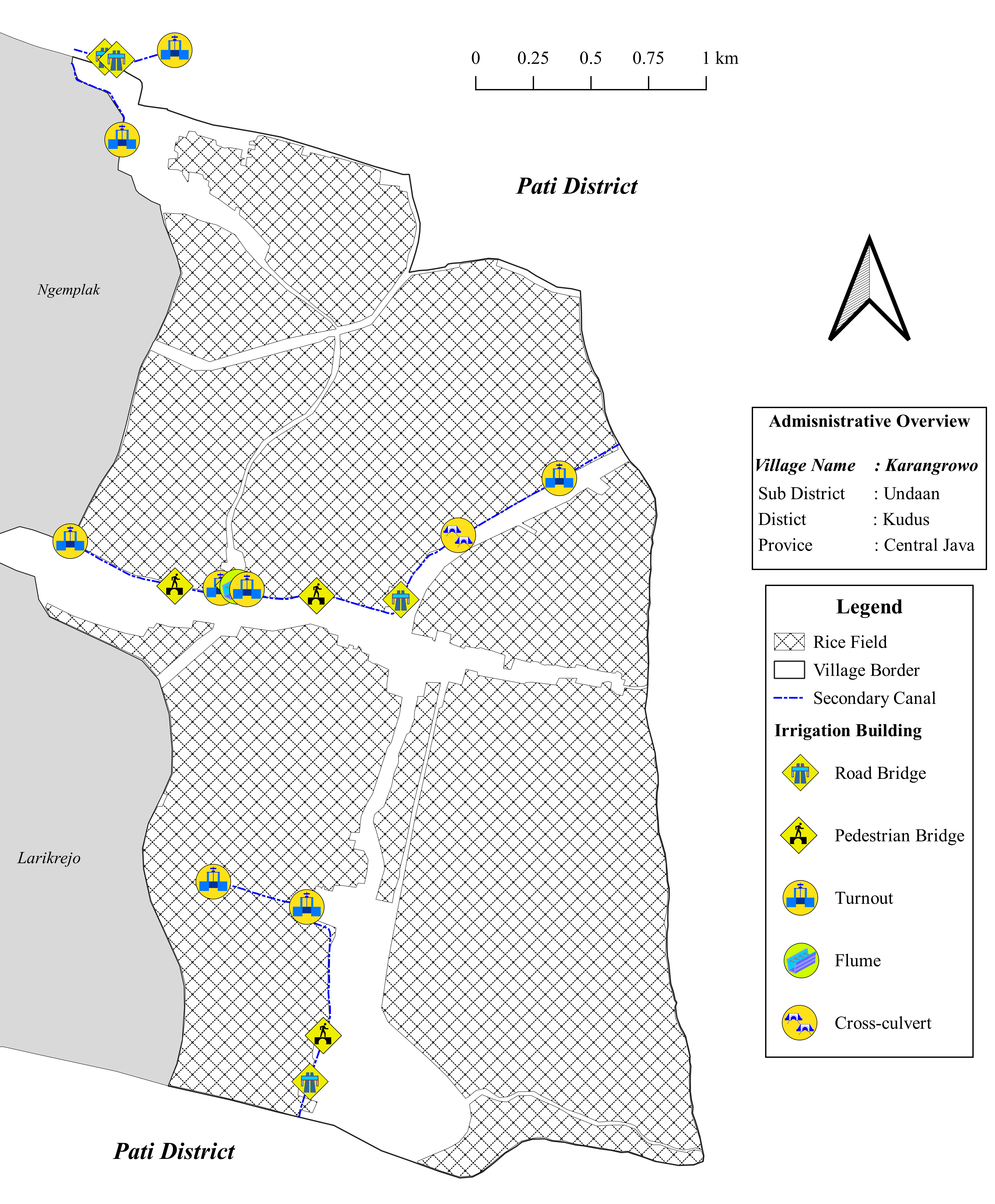

Supplement: Supplementary file 2 [file mmc2.zip › Supplementary data files/2. Irrigation Map/1. Kudus/8. Karangrowo.png]

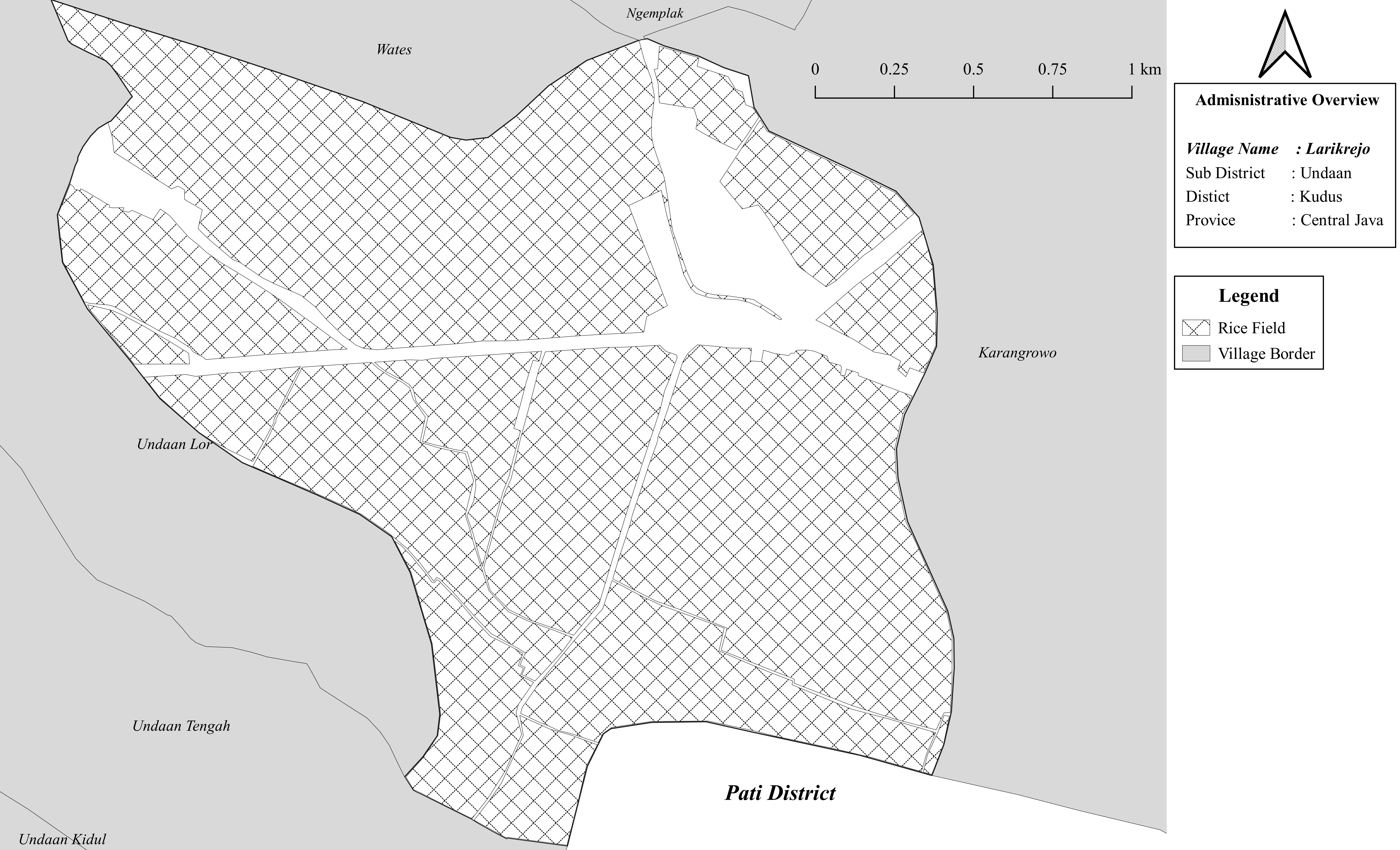

Supplement: Supplementary file 2 [file mmc2.zip › Supplementary data files/2. Irrigation Map/1. Kudus/9. Larikrejo.png]

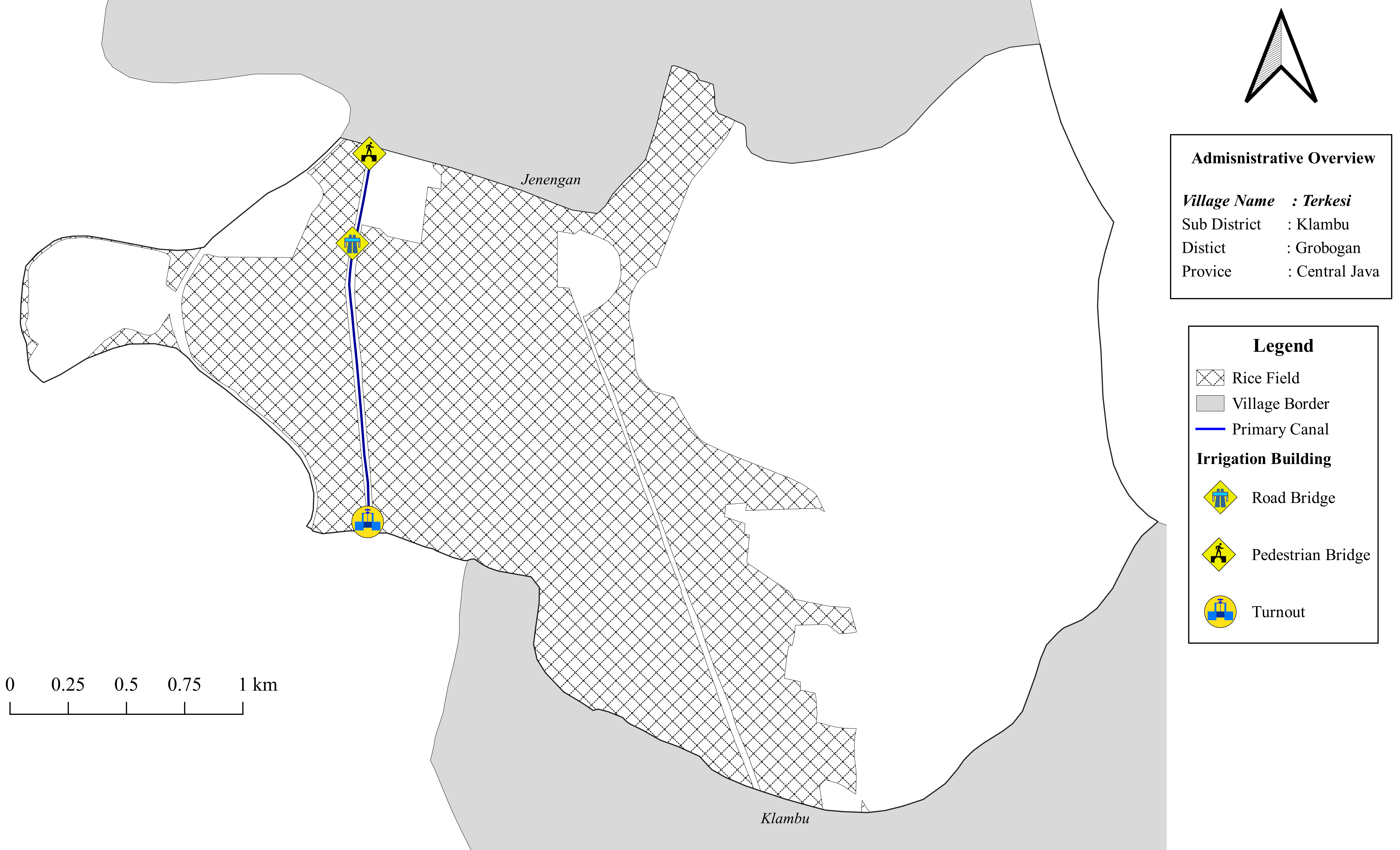

Supplement: Supplementary file 2 [file mmc2.zip › Supplementary data files/2. Irrigation Map/2. Grobogan/1. Terkesi.png]

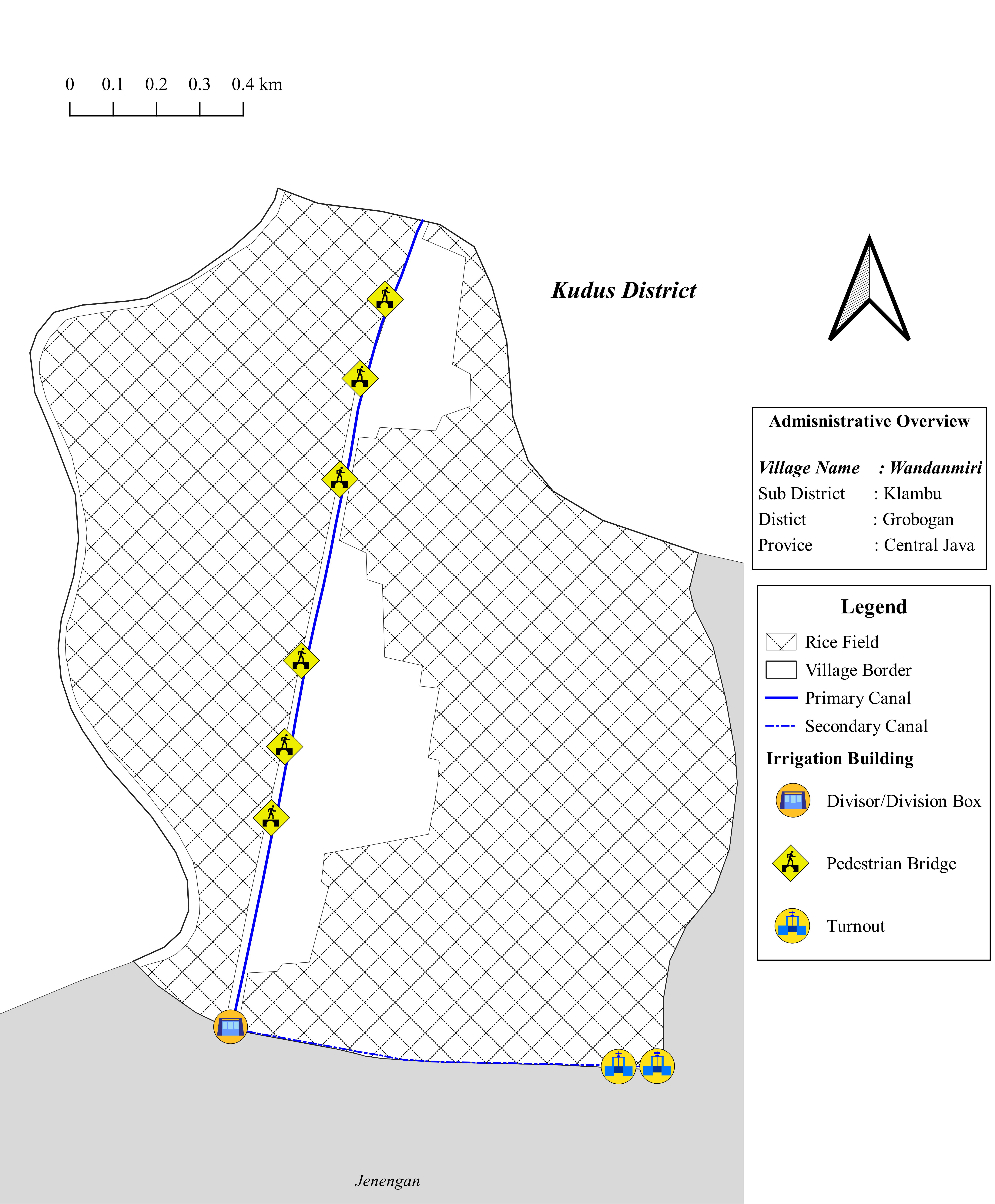

Supplement: Supplementary file 2 [file mmc2.zip › Supplementary data files/2. Irrigation Map/2. Grobogan/2. Wandanmiri.png]

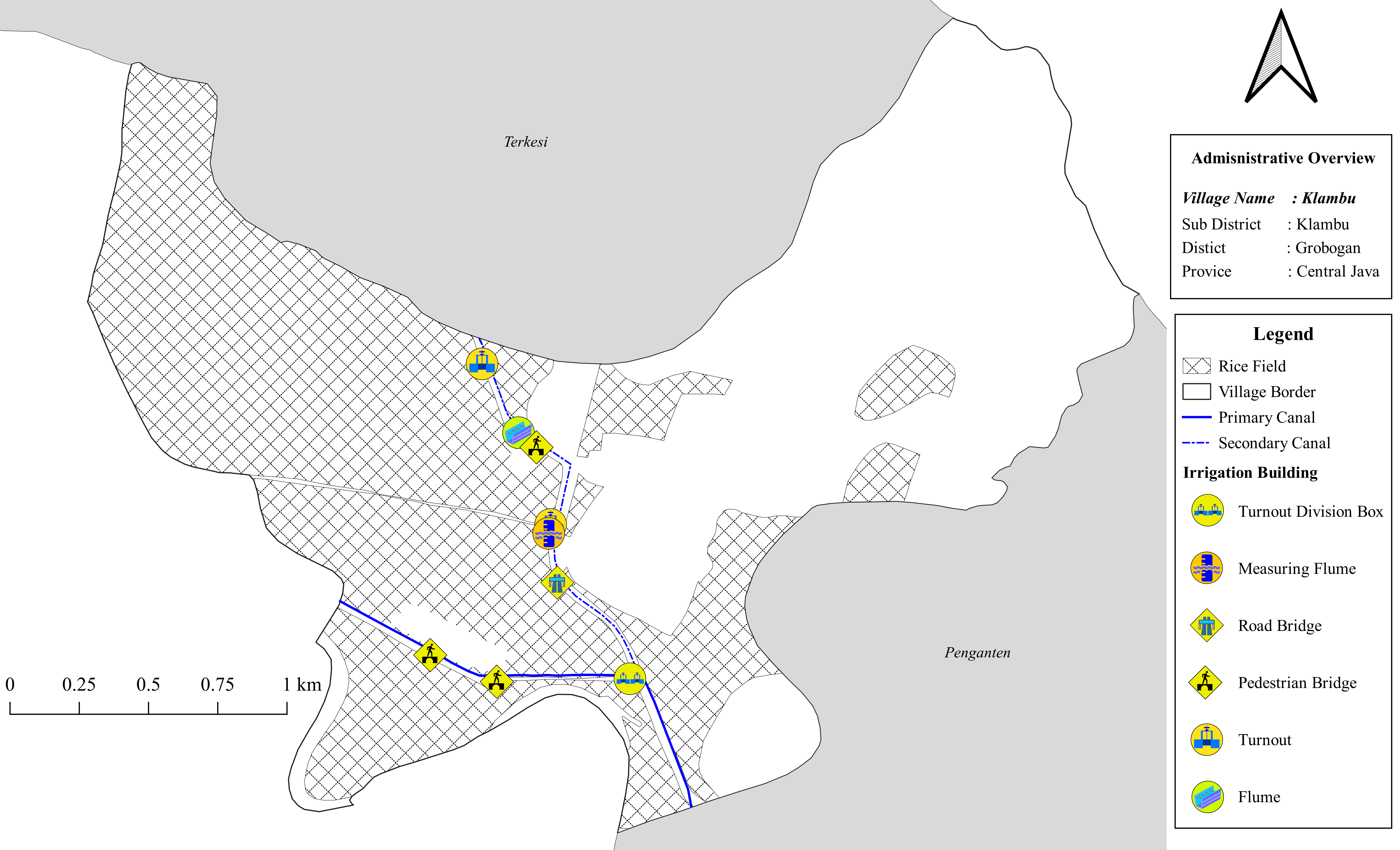

Supplement: Supplementary file 2 [file mmc2.zip › Supplementary data files/2. Irrigation Map/2. Grobogan/3. Klambu.png]

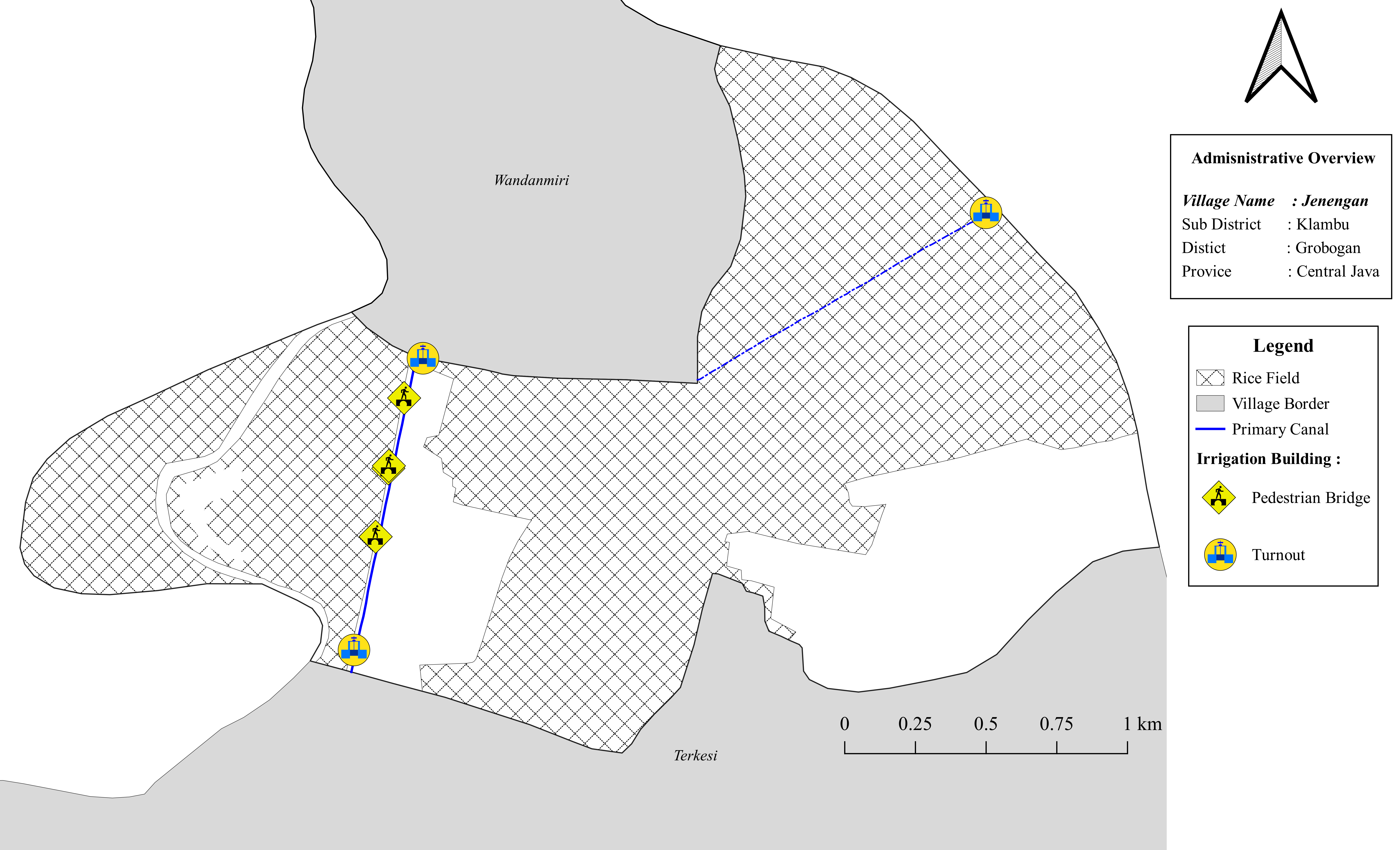

Supplement: Supplementary file 2 [file mmc2.zip › Supplementary data files/2. Irrigation Map/2. Grobogan/4. Jenengan.png]
